# Gut-Brain Axis: Unveiling the Interplay Between Diabetes Mellitus and Alzheimer's Disease

**DOI:** 10.7759/cureus.68083

**Published:** 2024-08-29

**Authors:** Haripriya S, Tamilanban T, Chitra Vellapandian

**Affiliations:** 1 Department of Pharmacology, Sri Ramaswamy Memorial (SRM) College of Pharmacy, SRM Institute of Science and Technology, Kattankulathur, IND; 2 Department of Pharmacy/Pharmacology, Sri Ramaswamy Memorial (SRM) College of Pharmacy, SRM Institute of Science and Technology, Kattankulathur, IND

**Keywords:** gut-brain axis, diabetes mellitus, alzheimer's disease, gut microbiota, hmgb1 pathway

## Abstract

The gut-brain axis (GBA) represents a complex bidirectional communication system linking the gastrointestinal tract with the CNS, influencing various physiological processes, including cognition. Emerging research suggests a significant interplay between diabetes mellitus (DM) and Alzheimer's disease (AD) mediated through this axis. DM, characterized by impaired insulin signaling and chronic inflammation, appears to exacerbate the pathology of AD. Key mechanisms include insulin resistance affecting neuronal function and promoting amyloid-beta accumulation and tau phosphorylation, hallmark features of AD. Additionally, dysbiosis of gut microbiota in DM may contribute to neuroinflammation and oxidative stress, further aggravating AD pathology. The gut microbiota can modulate systemic inflammation and metabolic dysfunction, potentially impacting AD progression in DM individuals. Understanding these interactions is crucial for developing targeted therapeutic strategies that address both DM and AD simultaneously. This abstract highlights the intricate relationship between metabolic disorders like DM and neurodegenerative conditions such as AD, emphasizing the role of the GBA as a pivotal area for future research and therapeutic interventions.

## Introduction and background

An assortment of prevalent endocrine disorders characterized by persistently elevated blood sugar levels is collectively called diabetes mellitus (DM) [1]. DM is brought on by either the pancreas producing insufficient insulin or the body's cells growing immune to its effects. Roughly 537 million adults globally, or 10.5% of the adult population, had DM as of 2021. About 90% of these cases are type 2 diabetes mellitus (T2DM). By 2045, one in eight individuals worldwide, or 783 million people, is predicted to have DM, a 46% increase above current projections. DM affects both men and women equally and is the seventh most prominent cause of death worldwide. Approximately US$760 billion is currently spent annually on DM-related healthcare globally. The typical indicators of unmanaged DM encompass increased urination (polyuria), persistent thirst, and unintended weight loss. Additionally, individuals may experience various general symptoms like fatigue, blurred vision, and genital discomfort caused by Candida infection. The long-term effect of DM increases the likelihood of cardiovascular disease by twofold, with approximately 75% of fatalities among DM individuals attributed to coronary artery disease. Additionally, DM also elevates the risk of stroke, peripheral artery disease, and AD, contributing to macrovascular complications and central nervous system (CNS) complications.

As people age, two age-related conditions that become more common and frequent are DM and AD. Research indicates that individuals diagnosed with DM have a higher probability of developing AD, and the presence of hyperinsulinemia and insulin resistance, which are characteristic features of DM, may contribute to cognitive impairment. Different degrees of nervous system dysfunction affect 60%-70% of DM. These conditions include diabetic neuropathy, which is characterized by reduced sensation or discomfort in the extremities; delayed stomach digestion; carpal tunnel syndrome; erectile dysfunction; and other peripheral nerve issues. Additionally, DM-related complications may interfere with CNS, possibly resulting in illnesses such as stroke and cognitive decline.

Elevated blood glucose levels over time contribute to the development of atherosclerosis, which hampers blood flow to the brain. Modifications to the blood-brain barrier (BBB) or the way small blood vessels in the brain function could also be responsible for CNS complications that could be connected to vascular dementia. When insulin attaches to its receptor's α-subunit in the brain, tyrosine kinase phosphorylates the β-subunit, starting a number of signaling pathways. One of these pathways is the neural Shc/mitogen-activated protein (MAP) kinase pathway. It improves the activation of genes necessary for the growth, maintenance, and repair of synapses and neurons. Additionally, it affects the hippocampal synaptic plasticity, which is essential for learning and memory functions.

Another mechanism is the binding of phosphoinositide 3-kinase by insulin receptor substrate (IRS)-1 and IRS-2. This relationship depends on synaptic plasticity, memory consolidation, recall, contextual memory extinction, and protection against Aβ-induced memory impairments. Additionally, this pathway promotes the production of nitric oxide, which is necessary for memory and learning. By phosphorylating N-methyl-D-aspartate glutamate receptors to increase calcium channel opening, regulating α-amino-3-hydroxy-5-methyl-4-isoxazolepropionic acid receptor internalization, and attracting gamma-aminobutyric acid (GABA) receptors to postsynaptic locations, insulin receptors also affect neurotransmission. These are the pathways mentioned above that are right now identified as DM-induced AD.

The high-mobility group box 1 (HMGB1) pathway is a crucial signaling mechanism in various physiological and pathological processes. HMGB1, a nuclear protein, can be released from cells under stress or injury and act as a damage-associated molecular pattern (DAMP) molecule, triggering inflammatory responses. The gut-brain axis (GBA), which is the term for the bidirectional communication between the gut and the brain, is greatly influenced by the HMGB1 pathway. The HMGB1 pathway has been implicated in several diseases, including neurodegenerative diseases like AD. Disruptions in the processing of insulin, defects in insulin receptors, or issues occurring after receptor activation can result in CNS issues, including AD, Parkinson’s disease, Huntington’s disease, various malignancies, migraine headaches, and schizophrenia [2].

Diabetes mellitus

Elevated blood sugar levels are a hallmark of DM, a metabolic disorder. It encompasses various types such as type 1, type 2, maturity-onset diabetes of the young, gestational diabetes, neonatal diabetes, and secondary forms due to other health conditions or medication use. The primary distinctions lie between type 1, which involves inadequate insulin production, and type 2, which involves either insufficient insulin action or production. Type 1 typically manifests in youth, while type 2 is commonly associated with adults who have prolonged elevated blood sugar due to lifestyle and dietary factors [3]. The two primary endocrine cell types found inside the pancreatic islets of Langerhans are beta cells, which produce insulin, and alpha cells, which produce glucagon. These cells use the body's glucose levels to control the amount of hormones they secrete. Abnormal glucose levels arise from an imbalance in glucagon and insulin secretion.

In DM, insulin is either deficient or its action is impaired, resulting in hyperglycemia. The hallmark of type 1 diabetes mellitus (T1DM) is the autoimmune destruction of beta cells, which leads to their complete loss and, as a result, a severe decrease in or absence of insulin. A progressive imbalance between insulin production and sensitivity characterizes the development of T2DM. Insulin resistance, often linked to factors like obesity and aging, plays a central role in T2DM pathogenesis, which can be estimated using the homeostatic model assessment for the insulin resistance index or the TyG index [4,5]. Globally, DM affects approximately one in 11 adults, with around 90% of cases being T2DM. T1DM typically emerges gradually from birth, peaking in incidence between ages four and six, and 10 and 14, with about 45% of cases occurring before age ten. The prevalence among individuals under 20 years old is approximately 2.3 per 1000. On the other hand, T2DM usually develops later in life, but rising obesity rates among adolescents have contributed to its emergence in younger age groups. In the US, T2DM affects about 9% of the general population, growing to approximately 25% among those over 65. Experts project a substantial increase in DM cases, from 415 to 642 million by 2040, particularly in populations transitioning from low- to middle-income brackets. Untreated DM commonly presents with classic symptoms such as increased urination (polyuria), thirst, and weight loss. Diabetic ketoacidosis is a dangerous medical disorder that primarily affects people with T1DM. At the same time, it can also develop in people with T2DM who have had the disease for a long time or when there is a major disruption in beta-cell activity. The condition is typified by an excess of ketone bodies being produced, which can cause symptoms like diminished consciousness in severe cases, acetone-like breath odor, nausea, vomiting, and stomach pain. Another emergency condition known as hyperosmolar hyperglycemic state is defined by excessive dehydration brought on by abnormally high blood sugar levels. This situation can lead to hypernatremia and altered mental status, which can ultimately result in coma [6].

Hypoglycemia, a side effect of insulin therapy for DM, can range in severity from less serious consequences like palpitations, sweating, and shaking to more serious ones like impaired judgment, disorientation, convulsions, coma, and, in rare cases, death. Hypoglycemia might lower the threshold for symptoms after repeated episodes; thus, mild symptoms might not show up before cognitive impairment does. Insulin plays multiple roles in regulating glucose metabolism. It inhibits glycogen breakdown and gluconeogenesis, promotes glucose uptake by fat and muscle cells, and facilitates glucose storage as glycogen. When blood glucose levels rise, which they frequently do after meals, beta cells in the pancreatic islets of Langerhans produce insulin, which is released into the bloodstream. Approximately two-thirds of the body's cells use it to absorb glucose from the bloodstream, which they then use for energy production, conversion into necessary chemicals, or storage for later use. Decreased blood glucose levels lead to reduced insulin secretion and the breakdown of glycogen into glucose. Glucagon, a hormone with opposing actions to insulin, primarily regulates this process.

Hyperglycemia, or high blood sugar levels, can contribute to inflammation in the GBA through various mechanisms. The GBA refers to the bidirectional communication between the gut and the brain, involving neural, hormonal, and immunological pathways. Hyperglycemia should significantly impact gut microbiota and contribute to neuroinflammation through several interconnected mechanisms. Elevated blood sugar levels alter the gut microbiota's makeup, resulting in dysbiosis associated with heightened gut inflammation. This inflammation can trigger immune responses, contributing to systemic inflammation along the GBA. Hyperglycemia is also linked to increased intestinal permeability, or "leaky gut," which allows bacteria, toxins, and other harmful substances to pass through the intestinal barrier and enter systemic circulation. These substances can activate immune cells and promote inflammation, potentially affecting the brain via the GBA. Persistent activation of immune responses in the intestines due to prolonged high blood sugar levels leads to the release of inflammatory cytokines and other compounds, further driving systemic inflammation.

Gut-based inflammation can impact neurological function directly via neural channels or indirectly through the circulatory system. This ongoing neuroinflammation, characterized by the production of inflammatory chemicals and immune cell activity within the brain, is linked to various neurological disorders. Hyperglycemia-induced inflammation in the gut may exacerbate neuroinflammation, impacting brain function and contributing to the development and progression of neurological conditions. Overall, hyperglycemia-induced inflammation in the gut can disrupt the balance of the GBA, causing systemic inflammation and potentially impacting neurological function and health. DM contributes to AD by disrupting the GBA via the HMGB1 pathway. This section will provide further details on how this mechanism influences AD.

Alzheimer's disease

**Figure 1:**
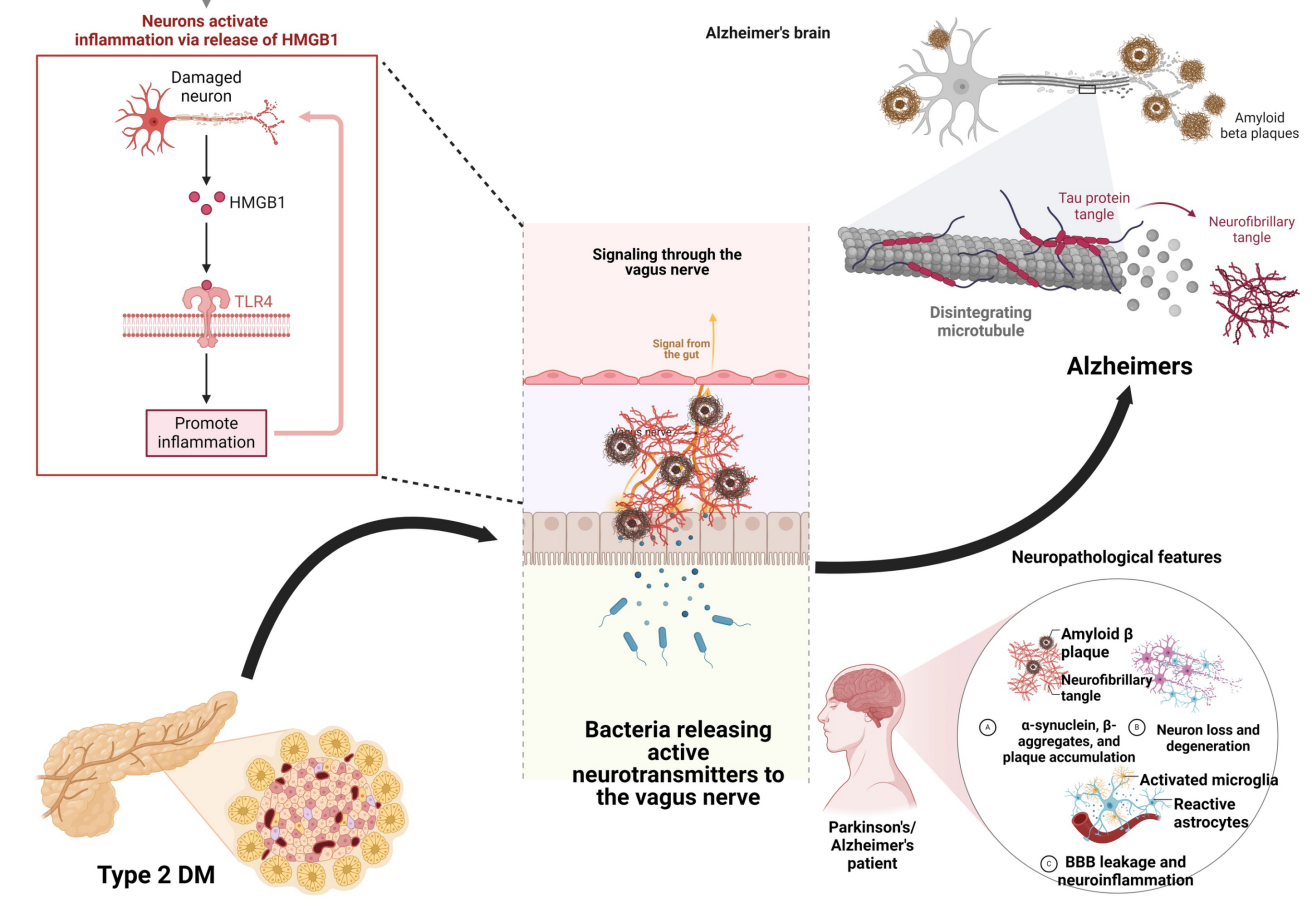
HMGB1 pathway interlinking diabetes and AD through GBA HMGB1: high-mobility group box 1; TLR4: toll-like receptor 4; DM: diabetes mellitus; BBB: blood-brain barrier; AD: Alzheimer's disease; GBA: gut-brain axis The figure is an original illustration of the author. Image credits: Haripriya S. Created by using Biorender.com

AD is a profoundly debilitating brain disorder predominantly affecting the elderly. It remains significantly underdiagnosed and undertreated, posing a major public health challenge. This disease, which was first recognized by Alois Alzheimer in 1906, is characterized by a progressive loss of memory, disorientation, and certain pathological characteristics such as senile plaques and neurofibrillary tangles. As the most prevalent form of dementia, AD affects at least 27 million people worldwide, accounting for 60%-70% of all dementia cases [7]. The impact of AD extends beyond the patients, severely affecting their families and imposing substantial financial burdens on society. The disease is marked by two primary lesions: senile plaques, which are extracellular deposits of β-amyloid protein (Aβ42), and neurofibrillary tangles, which are intraneuronal accumulations of phosphorylated P-tau (Figure 1).

Additionally, β-amyloid protein deposition can occur in the walls of capillaries, arteries, and arterioles, leading to amyloid cerebral angiopathy. This condition causes degeneration of vascular components, impairs blood flow, and increases the risk of intraparenchymal hemorrhages. Most of all, there is still much we do not understand regarding the disease's provenance, which appears to involve a delicate balance between genetic predispositions and environmental factors. The strongest genetic risks are associated with a version of the apolipoprotein E gene. However, other risk factors, such as previous head injuries, depression, and high blood pressure, also have a proven role.

Thereby, the progression of the disease largely consists of amyloid plaque formation, neurofibrillary tangles, and neural connections deterioration through a patient's brain. It can sometimes be years before the first symptoms are recognizable, with many experiencing prolonged periods that they would later describe as just what it is like to get older. The early symptoms could impact even the most intricate activities of daily living. Very frequently, mild cognitive impairment (MCI) has been described as a transitional stage between typical aging and dementia. One of the most prevalent forms is amnestic MCI. Amnestic MCI occurs when memory complaint is a dominant symptom and can often be considered an early stage of AD. In addition, memory impairment for the other episode types was insignificant [8]. If sleepiness comes with amnesic MCI, the probability of it being linked to AD is more than 90%.

AD originates from an abnormal accumulation of amyloid beta (Aβ), forming amyloid plaques externally and tau proteins internally, disrupting brain function and connectivity, and causing a gradual decline in cognitive abilities. This impaired protein clearance mechanism is influenced by age and regulated by brain cholesterol, and it is linked to other neurodegenerative conditions. The cholinergic hypothesis, the foundation of many drug treatments, suggests that AD results from diminished acetylcholine production due to the loss of cholinergic neurons in key brain regions like the limbic system and cerebral cortex. Additionally, the amyloid hypothesis posits that the deposition of extracellular Aβ is central to the disease [9], supported by the presence of the amyloid precursor protein gene on chromosome 21 and the early onset of AD symptoms in individuals with trisomy 21 (Down syndrome) [10]. A specific variant of apolipoprotein, apolipoprotein E4, significantly increases the risk of AD, as it is less effective in breaking down beta-amyloid, leading to its accumulation in the brain. In this section, we discussed the AD mechanism that GBA may be involved in. In this upcoming section, the GBA mechanism is explored. In this review, we looked up in detail the role of the HMGB1 pathway in developing DM-induced AD.

## Review

Gut-brain axis

The GBA refers to the bidirectional biochemical communication between the gastrointestinal (GI) tract and the CNS. The gut microbiota's functions in biochemical signaling between the GI and the CNS are called the "microbiota-GBA" in this context. Gut microbes, including bacteria, viruses, fungi, archaea, and their metabolites and associated compounds, are part of this two-way communication network. Understanding the relationship between the stomach and the brain has advanced recently, revealing a complex system that affects emotions, motivation, and health care professionals and regulates GI functions. This complex network, called the GBA, coordinates gut activity and links emotional and cognitive brain areas with different intestinal processes, including immune responses, digestion, reflexes, and hormone signaling. Neurological, immunological, and hormonal signals are combined to function the GBA and enable two-way communication. The hypothalamic-pituitary-adrenal (HPA) axis, autonomic nervous system (ANS), enteric nervous system (ENS), and CNS (which consists of the brain and spinal cord) are important constituents.

The process by which intestinal microbes colonize the gut, commonly known as the "second brain," mostly takes place in the early stages of development and is impacted by a number of variables, including feeding patterns, antibiotic exposure, gestational age, and delivery style. Throughout their various life stages, microbial communities change regarding their diversity, composition, and functions. The vagus nerve, the HPA axis, the immune system, and metabolites made by intestine microbes, such as short-chain fatty acids (SCFA), are just a few of the avenues by which these gut flora might affect the CNS. The human gastrointestinal system is home to the enteric microbiota. Although the makeup of microorganisms varies from person to person, healthy individuals generally have similar distribution and prevalence of different bacterial kinds in the intestinal area. Two-thirds of the microbiome comprises the main phyla, Firmicutes and Bacteroides. This microbial population is essential to the host's physiology and metabolism, supporting its general homeostasis and well-being throughout its existence.

Chemical substances released by the microbiome in the digestive system can significantly impact brain development, starting from infancy. One example is secondary bile acids produced by gut microbes, which may affect cognitive function. Changes in bile acid levels, such as an increase in harmful secondary bile acids and a decrease in primary bile acids, are observed in individuals with MCI and AD [11]. These observations suggest that gut flora may influence AD progression [11]. However, amidst the harmful effects of some secondary bile acids, tauroursodeoxycholic acid, a bile acid variant, shows promise for potentially benefiting the treatment of neurodegenerative diseases.

In addition to producing gut-derived peptides like glucagon-like peptide 1 (GLP-1), CCK, and peptide YY (PYY) and neuroactive substances like GABA and serotonin (5-hydroxytryptamine, 5-HT), the gut microbiota can also create regulatory components that may impact T and B cells. These byproducts can directly communicate with the brain by interacting with vagus nerve sensory pathways and local ENS cells. Additionally, they can trigger the HPA axis, which releases stress chemicals like cortisol. They might also affect the BBB's integrity, cause inflammation of neurons and stimulation of microglial cells, and aid in the emergence of illnesses affecting the CNS. Probiotics can improve cognitive function and the CNS by activating the vagus nerve. *Lactobacillus reuteri* supplementation improved synaptic plasticity in the ventral tegmental region after social interactions in a mouse model of autism spectrum disorders, and this effect was dependent on vagus nerve stimulation [12]. In addition to the gut microbiota, current research on the microbiota-GBA focuses on a number of important pathways, including the vagus nerve, the immunological and neuroendocrine systems, neurotransmitters, and metabolites. The vagus nerve, sometimes called the tenth cranial nerve, runs from the brain to the belly and controls internal organ processes like breathing, heart rate, and digestion. It consists of efferent (outgoing) and afferent (incoming) neurons that help send messages from the brain to many organs, including gut microbiota-influenced intestinal cells. The brain can effectively monitor the gastrointestinal environment thanks to this neuronal link.

Dextran sulfate sodium was used to induce intestinal inflammation in a rat model of AD, which activated the vagus nerve and led to cognitive loss. Vagotomies, which involve surgically severing the vagus nerve, particularly inhibited this signaling pathway, lowering amyloid-β levels and limiting Tau damage. Additionally, learning and memory-related cognitive abilities were restored by this intervention. Furthermore, caudal vagotomy mitigated neurodegeneration and related behavioral problems by preventing the transfer of α-synuclein nuclear lesions from the gut to the brain. Additionally, vagotomy demonstrated effectiveness in reducing symptoms of illnesses of the CNS, including anxiety and cognitive decline brought on by intestinal disorders. Neuroactive compounds interacting with the ANS include glutamic acid, 5-HT, and GABA. They can also affect the stomach vagal neurons. Researchers discovered a vagus nerve network that links the gut to the central amygdala nucleus in a rat model of anxiety. By blocking this pathway, the central amygdala's gene expression was changed, especially those linked to GABA, which had an antianxiety impact [13].

To connect with the brain and directly affect the vagus nerve, intestinal endocrine cells release hormones, including glutamate and 5-HT, which provide information about the nutritional state of the intestinal lumen. These cells also have receptors that can recognize metabolites and release GLP-1, PYY, and cholecystokinin (CCK). For instance, local stimulation of intestinal epithelial cells by GLP-1 might produce 5-HT, which inhibits the vagus nerve route. Since the vagus nerve is essential to microbiota-GBA, it is unclear exactly how gut flora and microbial metabolites affect vagal sensory pathways. More research is required to completely understand the intricate neural networks underlying the gut-microbiota-metabolite-brain axis. In summary, the vagus nerve functions as a two-way communication channel between the gut and the brain. It allows microbiota and their metabolites in the gut to influence brain function and vice versa, highlighting the importance of the GBA in health and disease.

To facilitate signal transmission across various systems, including the ANS, ENS, HPA axis, sympathoadrenal axis, and descending monoaminergic pathways, afferent neurons transmit signals inward, while efferent neurons carry signals outward. Afferent neurons play crucial roles in connecting neural pathways. These pathways and systems are highly interdependent and controlled by various neurohumoral variables. The intricate network of neurons that makes up the ENS is principally in charge of independently regulating gastrointestinal processes. It comprises two ganglionated plexuses that control gut motility (peristalsis), secretion, and absorption: the myenteric and submucosal plexuses. The ENS and the CNS can communicate through intestinofugal neurons, which transmit sensory data via primary afferent neurons along vagal pathways in gut-brain communication. The ANS directly impacts gut physiology through neurological connections that originate in the CNS. Additionally, the metabolites produced by the gut microbiota interact with one another through host cell detection, potentially influencing synaptic connections between the ANS and the gut. Furthermore, the ANS can impact gut epithelial mechanisms, potentially activating the immune system by directly modifying how gut immune cells respond to microbes or indirectly by altering microbial interactions with gut immune cells [14].

Afferent pathways, such as sensory fibers of the vagus nerve, can detect changes in the gut environment, including the presence of microbiota and their metabolites. Information about gut conditions, such as inflammation or the presence of certain nutrients or neurotransmitters produced by gut microbes, can be transmitted via these sensory fibers. Different pathways allow sensory neurons in the vagus nerve to communicate with immune cells and the microbiota. Some pathogenic microorganisms can directly activate these neurons when the gut barrier is breached. Nonetheless, the most advantageous gut flora indirectly affects the vagus nerve, frequently through enteroendocrine cells or enteric neurons. Serotonin might be involved in this mechanism. Sensitive neurons carrying numerous cytokine receptors probably pick up inflammatory cues generated by gut immune cells, like tumor necrosis factor and interleukin 1. These cytokine impulses are converted into brain signals, or neurograms, which, in turn, trigger the vagus nerve's efferent route, thereby controlling the actions of immune cells and inducing the "inflammatory reflex." Similar to the efferent pathway, the vagus nerve's motor fibers can send impulses from the brain to the stomach. These signals can influence gut motility, secretion of digestive enzymes, and even the composition of gut microbiota through mechanisms like peristalsis and regulation of gut immune responses.

The GBA pathway relies on a network of communication involving several key neurotransmitters, including GABA, dopamine, serotonin (5-HT), and noradrenaline. Moreover, substances like bile acids, SCFA, and neuroactive amino acids like tyramine and tryptophan have significant functions. Behavior, cognition, and the body's reaction to stress, anxiety, and fear are all significantly influenced by GABA. Psychiatric disorders like schizophrenia, autism, and depression are associated with low levels of GABA. According to studies on germ-free animals, the microbiota may impact the levels of circulating GABA, according to ongoing research into microbiota modulation. Certain Lactobacilli and specific strains of Bifidobacterium can also produce GABA [15]. The microbiota has been extensively studied for its role in regulating serotonin in the gut. Serotonin is essential to control mood, hunger, sleep patterns, and cognitive abilities. Selective serotonin reuptake inhibitors are commonly prescribed for treating depression by increasing serotonin levels in the brain [16]. Additionally, studies indicate that the gut microbiota may impact serotonin production through modifications to tryptophan absorption, which is a precursor amino acid [16].

Gut microbiota can directly and indirectly influence vagus nerve activity [17]. For example, microbes in the gut produce metabolites like SCFAs, which can activate vagal sensory fibers directly by binding to receptors on the vagal neurons. Additionally, the microbiota can affect gut immune responses, leading to the release of cytokines and other molecules by immune cells, which, in turn, can modulate vagus nerve function. This interplay between metabolites and immune modulation illustrates the complex ways in which the gut microbiota impacts vagal nerve activity. Gut-derived signals via the vagus nerve can travel to the brainstem and other brain areas, impacting mood, thought processes, and behavior, among other things. Neurotransmitters involved in mood and emotion regulation, such as dopamine and serotonin, can be affected by these signals in terms of their release in the brain. Treatments such as depression and epilepsy have been achieved with vagus nerve stimulation, demonstrating the importance of this channel in brain-gut communication. According to recent research, gut microbiota microorganisms can release amyloids and lipopolysaccharide (LPS). These compounds can potentially activate brain microglia, which, in turn, produce proinflammatory cytokines associated with the onset of AD.

The role of gut microbiota in AD is a complex and evolving area of research. While direct causality is still under investigation, several mechanisms have been proposed to explain how gut microbiota could contribute to AD. Dysbiosis, or a dysfunction in the microbes in the intestines, can have a major effect on neurodegenerative processes, such as those seen in AD, through several mechanisms. Dysbiosis often leads to increased production of proinflammatory chemicals and immune responses, contributing to systemic inflammation that may exacerbate neuroinflammation, a hallmark of AD, thereby accelerating neuronal damage and cognitive decline. Additionally, certain gut bacteria produce neurotoxic metabolites, such as amyloids and LPS, which can impair normal brain function and potentially contribute to the formation of amyloid-beta plaques and tau protein tangles characteristic of AD. Bowel accessibility, or "leaky gut," is another way dysbiosis might promote neurodegenerative processes by increasing the bloodstream's ability to carry bacterial toxins and inflammatory chemicals to the brain. Moreover, changes in the microbes in the gut can affect the synthesis of neurotransmitters that affect brain function, such as serotonin and GABA, which are partially produced by the gut flora. Finally, dysbiosis will affect the host immune system, leading to aberrant immune activation or dysfunction, contributing to neuroinflammation and neurodegeneration associated with AD. The current study aims to elucidate the specific mechanisms by which gut microbiota may influence the development and course of AD. Understanding these pathways could lead to novel therapeutic approaches targeting gut microbiota to potentially mitigate AD risk or progression. DM-induced AD causes dysregulation of GBA through the HMGB1 pathway. In the next section, the HMGB1 pathway is explored.

HMGB1 pathway

The HMGB1 pathway is a crucial signaling mechanism that is involved in various physiological and pathological processes. HMGB1, a nuclear protein, can be released from cells under stress or injury and act as a DAMP molecule, triggering inflammatory responses. A nuclear protein called HMGB1 is found in practically all cell types and is broadly dispersed. Besides its roles within cells, HMGB1 can also be released outside the cell, triggering innate immune responses such as chemotaxis and cytokine release. The protein includes three conserved cysteines (C23, C45, and C106) sensitive to redox changes; modifications to these cysteines influence the extracellular functions of HMGB1. The most prevalent nonhistone chromatin-associated proteins belong to the high-mobility group (HMG) superfamily. The HMGB, a special DNA binding domain these proteins possess, allows them to attach to non-B-type DNA structures such as hairpins, Z-DNA, and triplexes (H-DNA). Furthermore, DNA bending and looping can be induced by the HMGB domain [18].

**Figure 2:**
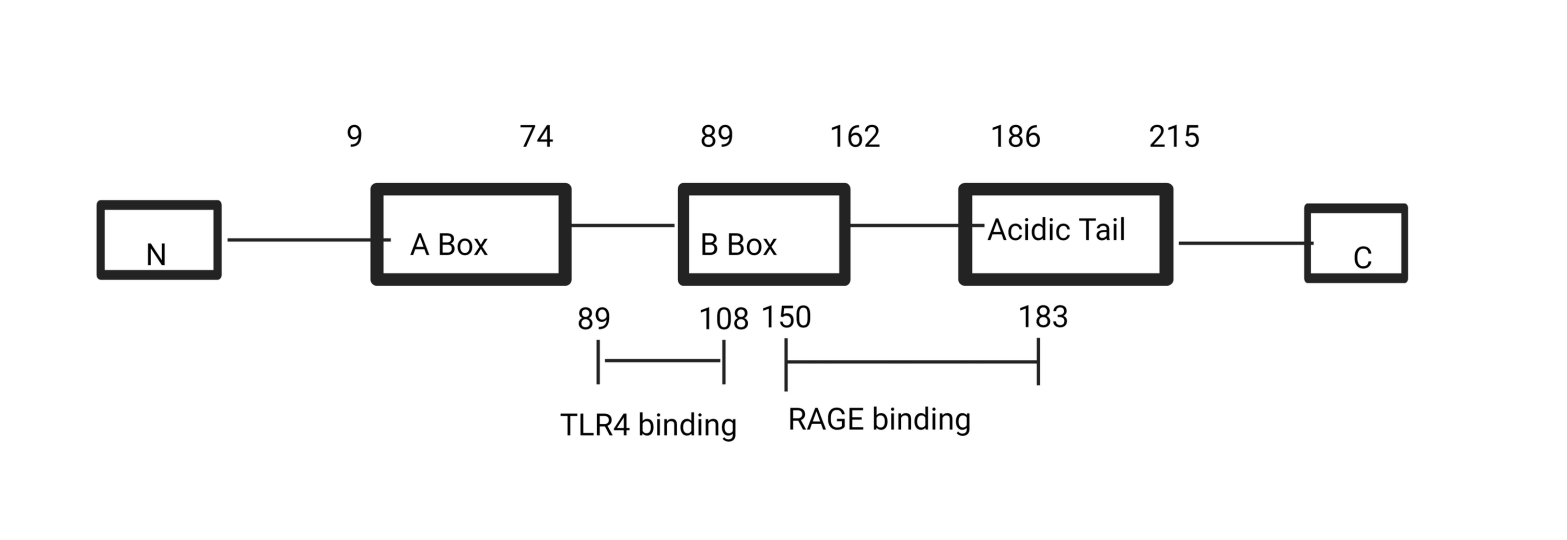
Schematic of the HMGB1 protein structure TLR4: toll-like receptor 4; RAGE: receptor for advanced glycation end-product; HMGB1: high-mobility group box 1 The figure is an original illustration of the author. Image credits: Haripriya S. Created by using Biorender.com

The HMG protein was originally discovered in the bovine thymus. The most common member of the HMGB family is HMGB1, found in 1973 by Goodwin et al. [19]. He was the first person to be identified as a member of the HMGB family. According to Bustin et al., the HMG family is categorized into three groups based on their functional characteristics: HMGA, HMGB, and HMGN. The HMGB group has three subtypes: HMGB1, HMGB2, and HMGB3. HMGB1 consists of three domains: an acidic tail composed of aspartic and glutamic acids (Figure 2) and two DNA-binding domains known as A box and B box. Both the A box and B box contain three α-helical structures that interact with DNA in a nonspecific manner. Two nuclear localization sequences for HMGB1 are found at the C-terminal tail (amino acids 179-185) and between the A and B boxes (amino acids 28-44) [20]. HMGB4, first recognized as a new member, is also connected to this family.

**Figure 3:**
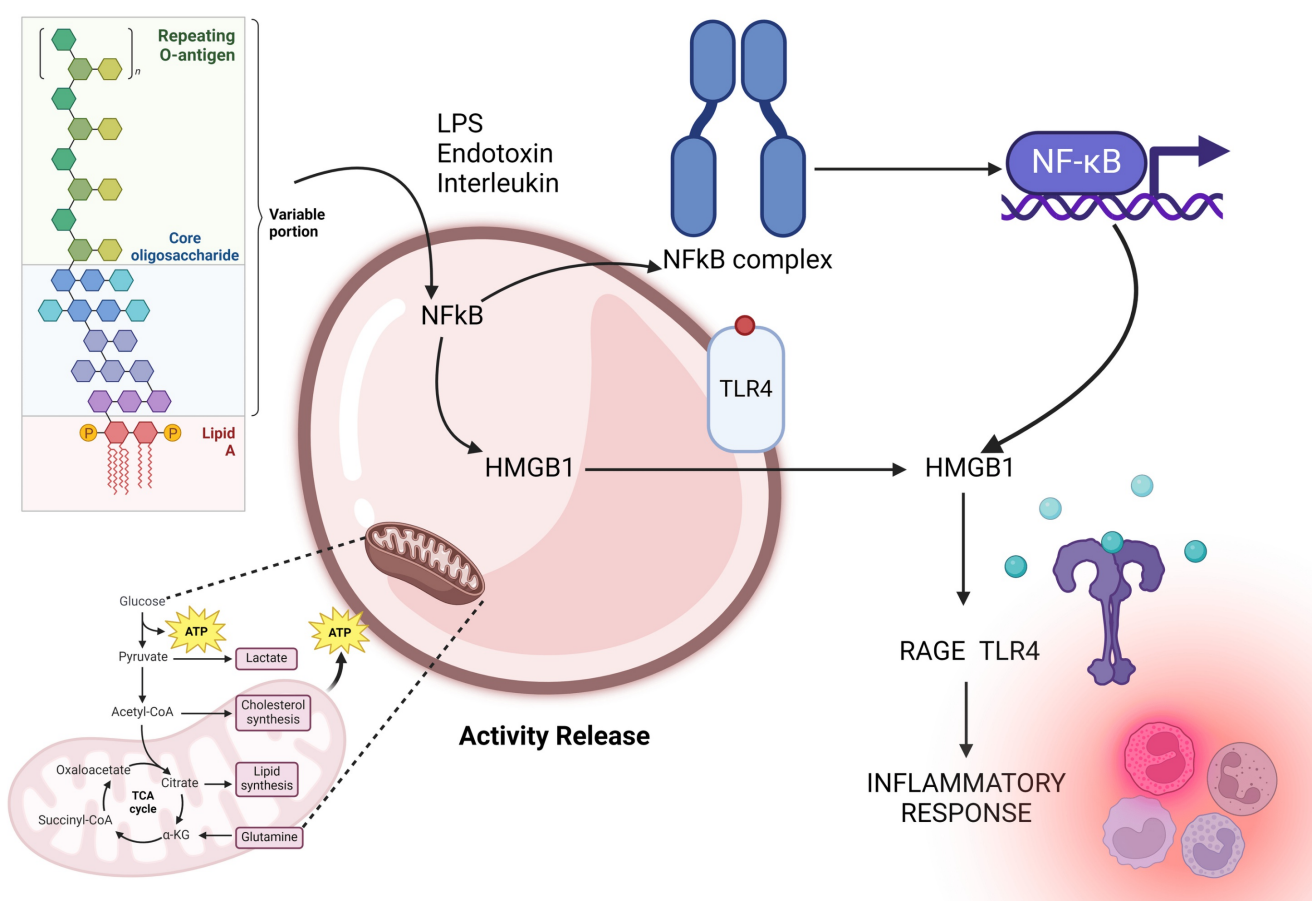
Endotoxin, LPS, and interleukins activate the nuclear factor kappa B, actively releasing HMGB1 pathway mediator inflammation in GBA LPS: lipopolysaccharide; NF-κB: nuclear factor kappa B; HMGB1: high-mobility group box 1; RAGE: receptor for advanced glycation end-product; TLR4: toll-like receptor 4; TCA: tricarboxylic acid; α-KG: alpha-ketoglutarate; GBA: gut-brain axis The figure is an original illustration of the author. Image credits: Haripriya S. Created by using Biorender.com

HMGB1 interacts with receptors, including toll-like receptor 4 (TLR4) and receptor for advanced glycation end product (RAGE) through the HMGB1 pathway. RAGE interacts with the molecule between proteins 150-183, whereas TLR4 attaches to the protein at sites 89-108 inside the HMGB1 B box [20]. Using the signaling pathways of MAP kinase and nuclear factor kappa-light-chain enhancer of activated B cells, these interactions activate proinflammatory cells and cause the overexpression of cytokines (Figure 3). The HMGB1 pathway has been implicated in several diseases, including inflammation, autoimmune disorders, and cancer. HMGB1 also influences AD and other disorders associated with cognitive decline. Understanding the intricacies of this pathway holds the potential for developing therapeutic interventions targeting inflammation and related conditions. According to recent research, HMGB1 binds to receptors like TLR4 and RAGE to serve as a mediator that promotes inflammation, contributing to neurodegenerative diseases like AD. Studies have shown that in DM-induced mouse models and DM patients, elevated blood sugar levels and insulin resistance lead to increased expression of HMGB1 and RAGE. In animal models of AD, as well as in the brains and cerebrospinal fluid of AD patients, elevated levels of HMGB1 have been seen [21]. The HMGB1-RAGE-TLR4 signaling pathway has been demonstrated in lab experiments to exacerbate hippocampus injury, which, in turn, contributes to the memory impairment seen in AD. Furthermore, HMGB1, RAGE, and TLR4 interactions have been linked to increased hippocampal neuron cell death under DM conditions. Additionally, these interactions have been connected to deficiencies in spatial memory, neuroinflammation, decreased insulin signaling, and increased formation of Aβ plaques.

Elevations in HMGB1 have been linked to injury to tissues and organs, as well as monocyte activation. Numerous illnesses, such as cancer, autoimmune disorders, and AD, are associated with elevated HMGB1 levels. Notably, patients with T2DM have been found to exhibit high plasma levels of HMGB1. Hyperglycemia and elevated HMGB1 expression are correlated. In AD, TLR4 expression is significantly elevated in the brain, contributing to immune responses, binding to amyloid peptides, and microglial phagocytosis [22]. During neuroinflammation induced by Aβ peptides, HMGB1 is localized in hippocampal neurons and plays a role in AD progression by activating RAGE/TLR4 signaling pathways [23]. Clinical studies have confirmed HMGB1 accumulation in both extracellular and intracellular brain regions of AD patients. The axis that mediates the connection between the gut and the brain depends heavily on the HMGB1 pathway. The immune system, the gut's ENS, the gut microbiota, and the CNS all play critical roles in this complex bidirectional communication system. Throughout the overall structure of GBA, HMGB1 released from gut epithelial cells, immune cells, or the microbiota can influence neuronal function and immune responses in the gut and the brain. HMGB1 mediates inflammation and immune activation, which can impact gut barrier integrity, neurotransmitter release, and neuroinflammation.

HMGB1 plays a crucial role in regulating intestinal barrier function, neuronal signaling, and immune activation, with significant implications for gastrointestinal and neurological health. By affecting the integrity of tight junctions between epithelial cells, HMGB1 can disrupt the intestinal barrier, and dangerous compounds and bacteria may be able to enter the circulation, which may result in neuroinflammation and systemic inflammation. In the CNS and ENS, HMGB1 can directly impact neuronal signaling by activating receptors such as RAGE and TLRs on neurons, which can alter neurotransmitter release, neuronal excitability, gastrointestinal motility, sensation, and visceral pain perception. Furthermore, HMGB1 acts as a potent proinflammatory mediator, activating immune cells locally in the gut and systemically, which can cause moderate gastrointestinal inflammation linked to neurological conditions like anxiety, depression, and neurodegenerative diseases. Additionally, the gut microbiota influences HMGB1 signaling; dysbiosis can lead to increased HMGB1 secretion and inflammation, while HMGB1 can affect gut microbiota composition by influencing immune responses and gut barrier integrity.

Comprehending the function of the HMGB1 pathway in the axis between the gut and the brain offers valuable perspectives on the processes that underlie the neurological exchange between the CNS and intestines. By focusing on this route, potential treatment strategies for diseases like irritable bowel syndrome, inflammatory bowel disease, and neuropsychiatric disorders that are marked by disruption at the GBA may develop. The pathways associated with DM and AD, including Aβ accumulation, inflammation, impaired insulin signaling, memory loss, and microglial activation, are influenced by the interaction of HMGB1, RAGE, and TLR4. Based on current studies, however, their specific involvement in DM-related cognitive impairment is still unclear. More research is necessary to find the essential molecular regulators in this setting and elucidate the processes by which HMGB1 signaling promotes dementia in DM. As a result, our review examines several perspectives and synthesizes current studies concerning the HMGB1-RAGE-TLR4 axis in AD associated with DM. In addition to suggesting that targeting HMGB1 signaling may be a viable strategy for treating AD in the setting of DM, this synthesis offers insightful avenues for future investigation.

DM-associated AD

The proposed mechanism linking DM with AD involves several interconnected pathways and factors. DM is marked by insulin resistance, where cells become less responsive to insulin. In the brain, insulin is crucial for neuronal survival, synaptic plasticity, and cognition; insulin resistance can impair insulin signaling pathways, leading to synaptic dysfunction, impaired neuronal metabolism, and cognitive decline. Additionally, DM disrupts glucose metabolism, resulting in chronic hyperglycemia that contributes to oxidative stress, mitochondrial dysfunction, and inflammation in the brain, factors implicated in AD pathology.

Elevated blood sugar levels in DM also result in the formation of AGEs, harmful compounds that accumulate in tissues, including the brain. AGEs can cross-link with proteins, altering their structure and function, which contributes to neurodegeneration and cognitive decline seen in AD. Furthermore, DM is associated with vascular complications such as endothelial dysfunction, microvascular damage, and impaired BBB function. These vascular changes can reduce cerebral blood flow, cause hypoxia, and decrease the delivery of nutrients and oxygen to the brain, all contributing to neurodegeneration.

DM also triggers a chronic low-grade inflammatory state characterized by elevated cytokine levels and activation of microglia, the brain’s immune cells. This chronic inflammation promotes amyloid-beta accumulation, tau hyperphosphorylation, and neuroinflammation, key pathological features of AD. Shared genetic risk factors and molecular pathways, including those involved in insulin signaling, lipid metabolism, and inflammation, further explain the concurrent emergence of both DM and AD. Dysregulation of these pathways links the two conditions, highlighting their interconnected nature. In summary, DM and AD share overlapping pathological mechanisms involving insulin resistance, glucose dysregulation, oxidative stress, inflammation, vascular dysfunction, and genetic factors. These interconnected pathways contribute to the increased risk and progression of AD in individuals with DM.

## Conclusions

Further research into the GBA, particularly the HMGB1 pathway, holds significant promise for developing targeted therapies for DM-induced AD. Understanding how gut microbiota influences HMGB1 activity and its extracellular roles in neuroinflammation could uncover novel therapeutic targets. By investigating the impact of gut dysbiosis on HMGB1-mediated immune responses, we may reveal new treatment strategies that address systemic metabolic dysregulation and neurodegeneration. This insightful exploration could ultimately pave the way for innovative approaches to mitigate cognitive decline in DM-related AD and improve outcomes for individuals affected by the complex interplay between DM and AD.
